# Prevalence of body-focused repetitive behaviors among the general population of Saudi Arabia: a cross-sectional study

**DOI:** 10.3389/fpsyt.2026.1704330

**Published:** 2026-02-03

**Authors:** Safiah A. Alamer, Hassan M. Alturaiki, Ali J. AlSaad, Ali M. Al Mousa, Amani A. Almutairi, Zahra S. Al-Sindi, Dalal M. Motabagani, Nouran D. AlShehri, Mariam M. Alshams

**Affiliations:** 1 Department of General Medicine, College of Medicine, King Faisal University, Al Ahsa, Saudi Arabia; 2 Department of Psychiatry, College of Medicine, King Faisal University, Al Ahsa, Saudi Arabia

**Keywords:** body-focused repetitive behaviors, hair-pulling behavior, nail-biting behavior, Saudi Arabia, skin-picking behavior

## Abstract

**Background:**

Body-focused repetitive behaviors (BFRBs)—including hair-pulling, skin-picking, and nail-biting—are increasingly recognized as behaviors associated with dermatologic, psychological, and social consequences. However, their prevalence in Middle Eastern populations remains underreported.

**Objective:**

To estimate the prevalence of BFRBs in the Saudi population and to describe their demographic distribution and clinical characteristics.

**Methods:**

A cross-sectional, community-based survey was conducted between June 2023 and July 2024. A total of 740 adults (aged 18–73 years) completed an online questionnaire incorporating the Habit Questionnaire, a brief five-item self-report screening tool assessing repetitive grooming behaviors, including type, frequency, duration, and impact. Study-defined operational criteria classified behaviors occurring ≥5 times per day for at least four weeks together with functional impairment, injury, or medical attention. Statistical analyses included descriptive summaries, Chi-square tests, and logistic regression.

**Results:**

The prevalence of BFRB-related behaviors meeting study-defined thresholds was 29.1%. Hair-pulling behavior was reported by 19.1% of participants, skin-picking by 13.4%, and nail-biting by 4.3%. Younger age was strongly associated with these behaviors, with prevalence reaching 47.2% in the 18–20-year group and decreasing to 10.1% in participants older than 50 years (p = 0.001). Most affected individuals described symptoms persisting beyond 12 months and reported complications including interference with daily functioning, injuries, and permanent scarring. Only 5.5% had sought medical care. In multivariable analysis, age remained the strongest predictor across all behavior types; female gender was associated with higher odds of hair-pulling and lower odds of nail-biting, while educational level showed no significant association.

**Conclusion:**

BFRBs, defined by study-specific behavioral thresholds, are common in Saudi Arabia, particularly among younger adults. Gender differences varied by behavior subtype. Despite chronicity and reported physical consequences, few individuals sought medical care. These findings highlight the need for early recognition, increased public awareness, and integration of BFRB screening into primary and mental health services.

## Introduction

Body-focused repetitive behaviors (BFRBs) are defined as repeated, unintentional, and uncontrollable grooming habits that focus on the body. These behaviors include biting, picking, or chewing various body parts, such as nails (nail-biting behavior), skin (skin-picking behavior), or hair (hair-pulling behavior) (1, 2). Previously considered harmless nervous habits, BFRBs are now recognized for their significant physical, emotional, and social consequences (3). For instance, they can lead to severe dermatological damage, including alopecia and tissue injury (4, 5).

The Diagnostic and Statistical Manual of Mental Disorders, Fifth Edition (DSM-5), classifies trichotillomania, excoriation disorder, and severe cases of nail-biting as obsessive–compulsive and related disorders due to their shared phenomenology and underlying behavioral patterns (1). These disorders are characterized by impulse-control difficulties, where individuals struggle to resist engaging in these behaviors despite negative consequences (3). Impulse-control problems are also prevalent in conditions such as Tourette syndrome, attention-deficit/hyperactivity disorder (ADHD), and oppositional defiant disorder (ODD), increasing the likelihood of engaging in repetitive grooming behaviors (3). Additionally, individuals with psychiatric conditions involving emotional-regulation difficulties—such as depression and anxiety—may use repetitive grooming behaviors as maladaptive coping mechanisms (6). Studies have shown a higher prevalence of skin-picking behaviors among individuals with severe depression (14.4%) compared with those with mild depressive symptoms (2.1–6.4%) (7).

BFRBs typically emerge in late childhood or adolescence (8). Community-based studies have reported varying prevalence rates among youth: 10.5% for hair-pulling, 5–29% for nail-biting, and 10–40% for skin-picking (9). A 2022 retrospective cohort study conducted among adolescents aged 9–17 found that BFRB symptoms intensified during activities such as studying, watching TV, or experiencing feelings of boredom (1). Another study of 318 participants found the distribution of BFRBs as follows: 6% for onychophagia, 6% for cheek or lip biting, 14% for dermatillomania, and 2% for trichotillomania (2). Several studies also suggest that females may be more prone to BFRBs than males (3).

A study conducted in three medical colleges in Karachi (age range: 18–27 years) found that 22% of students reported experiencing BFRBs. Among them, hair pulling/hair manipulation was the most prevalent (13.3%), followed by skin picking (9.0%) and nail biting (6.2%). The study also showed a higher prevalence in females (13.9%) than males (8.1%) (8). Another study involving 2,641 participants found that 59.5% of those with BFRBs were female (n = 1,839; 69.3%), whereas males accounted for 30.6% (n = 807) (10).

The prevalence of BFRB-like behaviors in Saudi Arabia remains unknown, presenting a significant gap in knowledge. This study aims to address this gap by assessing the prevalence of these behaviors in the Saudi population and identifying contributing factors. The findings will be crucial in developing effective treatment strategies and implementing prevention programs, including awareness campaigns. Despite their impact, many individuals with repetitive grooming behaviors fail to seek treatment, emphasizing the need for increased recognition and early intervention efforts. Although DSM-5 disorders are referenced in the background, this study does not establish clinical diagnoses and instead evaluates repetitive behaviors based on study-defined criteria.

## Methods

### Study design and setting

This cross-sectional study was carried out in Saudi Arabia between June 2023 and July 2024. The target population consisted of Saudi adults aged 18 years and older. Participants who completed the survey and provided consent were included, while responses missing essential information were excluded.

### Data collection tool and procedure

Data collection was performed using an online survey that incorporated the Habit Questionnaire, a brief five-item self-report instrument developed to provide a standardized assessment of the frequency, duration, and functional consequences of repetitive grooming behaviors. The Habit Questionnaire has demonstrated moderate test–retest reliability (r = 0.69, p < 0.001) and has been used in prior epidemiological studies examining body-focused repetitive behaviors (8). In this study, the instrument was used to assess engagement in hair-pulling, skin-picking, nail-biting, and related behaviors, along with their chronicity and associated impairment. The questionnaire was distributed electronically across diverse regions of Saudi Arabia to maximize accessibility. Participation was voluntary, informed consent was obtained electronically, and all responses were kept confidential with access restricted to the study investigators.

The questionnaire comprised two major sections. (A) Sociodemographic characteristics included age, gender, and educational attainment, providing the baseline descriptive profile of the study population. (B) The behavioral assessment addressed the presence of hair-pulling, nail-biting, and skin-picking behaviors. Participants were asked whether they engaged in these behaviors, the frequency and duration of such behaviors, and whether these actions interfered with their daily activities or led to injuries or permanent scarring. This study did not collect formal psychiatric or medical history variables.

### Operational definition of BFRBs

For classification purposes, participants were identified as having a BFRB if they reported engaging in at least one repetitive grooming behavior five or more times per day for a minimum duration of four weeks. In addition to this threshold, at least one of the following criteria had to be fulfilled: the behavior interfered with daily functioning, caused physical injury, led to medical consultation, or resulted in a medical recommendation to stop the behavior. These criteria represent study-defined thresholds and do not correspond to formal DSM-5 diagnostic criteria.

### Statistical analysis

Data were analyzed using SPSS, version 26.0 (SPSS Inc., Chicago, IL, USA). Descriptive statistics were applied to summarize demographic variables and the prevalence of BFRBs. Frequency distributions were generated for sociodemographic characteristics, types of BFRBs, behavioral frequency and duration, and consultation with healthcare providers. Associations between BFRB prevalence and demographic variables, including gender, age, and educational level, were examined using crosstabulation and Pearson’s Chi-square test. For small sample sizes, the exact probability test was applied. Statistical significance was defined as a p-value < 0.05.

### Ethical considerations

The study protocol was reviewed and approved by the Ethics Committee of King Faisal University (Approval No.: ETHICS1,590). All participants provided informed consent, and the principles of confidentiality and anonymity were strictly maintained throughout the study.

## Results

A total of 740 participants were included in the analysis. The mean age was 35.3 years (SD ±14.9), with a distribution ranging from 18 to over 50 years. The largest proportion of the cohort was within the 21–25-year age group (191 participants, 25.8%), followed by those older than 50 years (138, 18.6%) and the 41–50-year category (117, 15.8%). Participants aged 31–39 years accounted for 113 (15.3%), while those aged 18–20 years comprised 108 (14.6%). The smallest age group represented was 26–30 years, with 73 individuals (9.9%). Specifically, **Table 1** summarizes the sociodemographic characteristics of the study participants, and **Table 2** presents the gender-wise characteristics of body-focused repetitive behaviors.

**Table 1:** Socio-demographic characteristics of study participants (n=740).

Variable	Category	N (%)
Age (years)	18–20	108 (14.6%)
	21–25	191 (25.8%)
	26–30	73 (9.9%)
	31–39	113 (15.3%)
	41–50	117 (15.8%)
	> 50	138 (18.6%)
Gender	Male	293 (39.6%)
	Female	447 (60.4%)
Educational level	Below secondary	32 (4.3%)
	Secondary	179 (24.2%)
	University	485 (65.5%)
	Post-graduate	44 (5.9%)

**Table 2:** Characteristics of body-focused repetitive behaviors (BFRBs) among study participants by gender.

Variable		Hair pulling N (%)			Hair manipulation N (%)			Nail biting N (%)			Cheek/lip/mouth chewing N (%)			Other area biting N (%)		
		Total	Male	Female	Total	Male	Female	Total	Male	Female	Total	Male	Female	Total	Male	Female
Presence		66 (8.9%)	17 (5.8%)	49 (11.0%)	275 (37.2%)	78 (26.6%)	197 (44.1%)	124 (16.8%)	56 (19.1%)	68 (15.2%)	199 (26.9%)	55 (18.8%)	144 (32.2%)	82 (11.1%)	32 (10.9%)	50 (11.2%)
Frequency	<5/day	28 (42.4%)	7 (41.2%)	21 (42.9%)	91 (33.1%)	26 (33.3.3%)	65 (33.0%)	44 (35.5%)	20 (35.7%)	24 (35.3%)	95 (47.7%)	24 (43.6%)	71 (49.3%)	24 (29.3%)	10 (31.3%)	14 (28.0%)
	>5/day	20 (30.3%)	7 (41.2%)	13 (26.5%)	135 (49.1%)	35 (44.9%)	100 (50.8%)	32 (25.8%)	14 (25.0%)	18 (26.5%)	74 (37.2%)	22 (40.0%)	52 (36.1%)	31 (37.8%)	9 (28.1%)	22 (44.0%)
	<4/week	18 (27.3%)	3 (17.6%)	15 (30.6%)	49 (17.8%)	17 (21.8%)	32 (16.2%)	48 (38.7%)	22 (39.3%)	26 (38.2%)	30 (15.1%)	9 (16.4%)	21 (14.6%)	27 (32.9%)	13 (40.6%)	14 (28.0%)
Duration	<4 weeks	10 (15.2%)	2 (11.8%)	8 (16.3%)	30 (10.9%)	6 (7.7%)	24 (12.2%)	18 (14.5%)	7 (12.5%)	11 (16.2%)	13 (6.5%)	3 (5.5%)	10 (6.9%)	11 (13.4%)	5 (15.6%)	6 (12.0%)
	4–12 month	11 (16.7%)	4 (23.5%)	7 (14.3%)	39 (14.2%)	9 (11.5%)	30 (15.2%)	7 (5.6%)	5 (8.9%)	2 (2.9%)	27 (13.6%)	9 (16.4%)	18 (12.5%)	8 (9.8%)	3 (9.4%)	5 (10.0%)
	>12 month	45 (68.2%)	11 (64.7%)	34 (69.4%)	206 (74.9%)	63 (80.8%)	143 (72.6%)	99 (79.8%)	44 (78.6%)	55 (80.9%)	159 (79.9%)	43 (78.2%)	116 (80.6%)	63 (76.8%)	24 (75.0%)	39 (78.0%)
Interfered with activity		27 (40.9%)	3 (17.6%)	24 (49.0%)	44 (16.0%)	12 (15.4%)	32 (16.2%)	30 (24.2%)	14 (25.0%)	16 (23.5%)	27 (13.6%)	3 (5.5%)	24 (16.7%)	21 (25.6%)	8 (25.0%)	13 (26.0%)
Caused injury		26 (39.4%)	6 (35.3%)	20 (40.8%)	42 (15.3%)	17 (21.8%)	25 (12.7%)	66 (53.2%)	29 (51.8%)	37 (54.4%)	82 (41.2%)	23 (41.8%)	59 (41.0%)	37 (45.1%)	8 (25.0%)	29 (58.0%)
Permanent scar/damage		16 (24.2%)	4 (23.5%)	12 (24.5%)	28 (10.2%)	12 (15.4%)	16 (8.1%)	34 (27.4%)	14 (25.0%)	20 (29.4%)	24 (12.1%)	5 (9.1%)	19 (13.2%)	23 (28.0%)	4 (12.5%)	19 (38.0%)
Noticeable hair loss		44 (66.7%)	11 (64.7%)	33 (67.3%)	98 (35.6%)	25 (32.1%)	73 (37.1%)	–	–	–	–	–	–	–	–	–
Sought medical attention		6 (9.1%)	0 (0.0%)	6 (12.2%)	–	–	–	–	–	–	–	–	–	–	–	–

- Not applicable.

Participants could report more than one behavior.

With respect to gender distribution, females constituted the majority of the study population (447, 60.4%), whereas males accounted for 293 (39.6%). Furthermore, in terms of educational attainment, most participants had completed university education (485, 65.5%). Secondary-level education was reported by 179 participants (24.2%), and 44 individuals (5.9%) held postgraduate qualifications. A smaller subgroup of 32 participants (4.3%) had attained below-secondary education.

Hair pulling was reported by 66 participants (8.9%), with a higher prevalence among females (11.0%) compared to males (5.8%). Among those affected, 20 participants (30.3%) engaged in hair pulling more than five times per day, 28 (42.4%) reported fewer than five times per day, and 18 (27.3%) reported fewer than four times per week. With regard to duration, 45 individuals (68.2%) indicated the behavior had persisted for more than 12 months, 11 (16.7%) for four weeks to 12 months, and 10 (15.2%) for less than four weeks. Noticeable hair loss was reported by 44 participants (66.7%), while 27 (40.9%) stated that the behavior interfered with daily activities. Injury related to hair pulling was noted in 26 participants (39.4%), and 16 (24.2%) reported permanent scarring or damage. A small subgroup of six individuals (9.1%) sought medical attention for this behavior.

Hair manipulation was more common, reported by 275 participants (37.2%), including 197 females (44.1%) and 78 males (26.6%). Among these, 135 (49.1%) engaged in the behavior more than five times daily, 91 (33.1%) less than five times daily, and 49 (17.8%) less than four times per week. Most participants (206, 74.9%) reported a duration exceeding 12 months, while 39 (14.2%) had engaged in the behavior for four weeks to 12 months, and 30 (10.9%) for less than four weeks. Noticeable hair loss occurred in 98 participants (35.6%), and 44 (16.0%) indicated interference with daily activities. Injuries were reported by 42 participants (15.3%), and permanent scarring was present in 28 (10.2%).

Nail biting was reported by 124 participants (16.8%), with slightly higher prevalence among males (19.1%) than females (15.2%). Frequency analysis showed that 32 participants (25.8%) engaged in nail biting more than five times per day, 44 (35.5%) less than five times per day, and 48 (38.7%) less than four times per week. A large proportion of individuals (99, 79.8%) had engaged in the behavior for more than 12 months, while 7 (5.6%) reported four weeks to 12 months, and 18 (14.5%) less than four weeks. Interference with daily functioning was reported by 30 participants (24.2%), while injuries were noted in 66 (53.2%) and permanent scarring in 34 (27.4%).

Cheek, lip, or mouth chewing was reported by 199 participants (26.9%), with higher prevalence among females (32.2%) than males (18.8%). Of these, 74 (37.2%) engaged in the behavior more than five times daily, 95 (47.7%) less than five times daily, and 30 (15.1%) less than four times per week. The majority (159, 79.9%) reported engaging in the behavior for longer than 12 months, while 27 (13.6%) reported four weeks to 12 months, and 13 (6.5%) less than four weeks. A total of 27 participants (13.6%) indicated interference with daily activities, while 82 (41.2%) reported injuries and 24 (12.1%) reported permanent scarring or damage.

Other area biting was reported by 82 participants (11.1%), with a similar prevalence among females (11.2%) and males (10.9%). Among those affected, 31 participants (37.8%) engaged in the behavior more than five times per day, 24 (29.3%) less than five times per day, and 27 (32.9%) fewer than four times per week. Regarding duration, 63 participants (76.8%) indicated the behavior had persisted for more than 12 months, while 8 (9.8%) reported four weeks to 12 months, and 11 (13.4%) reported a duration of less than four weeks. Interference with daily functioning was reported by 21 participants (25.6%), injuries by 37 (45.1%), and permanent scarring by 23 (28.0%).

**Table 3** presents the prevalence of hair-pulling, skin-picking, and nail-biting behaviors based on the study’s operational criteria, as well as the overall proportion of participants meeting these behavioral thresholds. Hair-pulling behavior was reported by 141 participants (19.1%), with a significantly higher prevalence among females (23.7%) compared with males (11.9%) (p = 0.001). Skin-picking behavior was reported by 99 participants (13.4%), also more frequent among females (15.7%) than males (9.9%) (p = 0.024). Nail-biting behavior was identified in 32 participants (4.3%), with comparable rates between males (4.8%) and females (4.0%) (p = 0.623). When considering the presence of any repetitive grooming behavior that met the study-defined frequency and impairment criteria, 215 participants (29.1%) fulfilled at least one behavioral threshold, with a higher proportion among females (33.8%) than males (21.8%) (p = 0.001).

**Table 3:** Prevalence and gender-wise distribution of body-focused repetitive behaviors (BFRBs) among study participants.

Behavior	Total n (%)	Male n (%)	Female n (%)	p-value
Hair-pulling behavior	141 (19.1%)	35 (11.9%)	106 (23.7%)	0.001*
Nail-biting behavior	32 (4.3%)	14 (4.8%)	18 (4.0%)	0.623
Skin-picking behavior	99 (13.4%)	29 (9.9%)	70 (15.7%)	0.024*
Any BFRB (+ve)	215 (29.1%)	64 (21.8%)	151 (33.8%)	0.001*

*P < 0.05 (significant).

**Table 4** illustrates the distribution of BFRBs according to age and educational level. Younger participants exhibited significantly higher rates of BFRBs compared to older age groups (p = 0.001). The prevalence of BFRBs was 47.2% in the 18–20-year group, 44.5% in those aged 21–25 years, and 43.8% among participants aged 26–30 years. In contrast, the prevalence decreased with increasing age, reaching 17.7% among those aged 31–39 years, 11.1% among those aged 41–50 years, and 10.1% among participants older than 50 years.

**Table 4:** Age- and education-related differences in BFRBs among study participants.

Variable	Category	BFRB – No n (%)	BFRB – Yes n (%)	p-value
Age (years)	18–20	57 (52.8%)	51 (47.2%)	0.001^*
	21–25	106 (55.5%)	85 (44.5%)	
	26–30	41 (56.2%)	32 (43.8%)	
	31–39	93 (82.3%)	20 (17.7%)	
	41–50	104 (88.9%)	13 (11.1%)	
	> 50	124 (89.9%)	14 (10.1%)	
Educational level	Below secondary	25 (78.1%)	7 (21.9%)	0.591
	Secondary	127 (70.9%)	52 (29.1%)	
	University	339 (69.9%)	146 (30.1%)	
	Post-graduate	34 (77.3%)	10 (22.7%)	

BFRBs, body-focused repetitive behaviors.

^Exact probability test.

*P < 0.05 (significant).

With regard to education, no statistically significant differences were observed (p = 0.591). The prevalence of BFRBs was 21.9% among individuals with below-secondary education, 29.1% among those with secondary education, 30.1% in university graduates, and 22.7% among participants with postgraduate education.

Multivariable logistic regression analysis (**Table 5**) demonstrated that younger age was consistently associated with higher risk across all outcomes. For each year increase in age, the odds of having any BFRB decreased by 6% (OR = 0.94, 95% CI: 0.93–0.96, p = 0.001). Similarly, younger age was significantly associated with Skin-picking behavior (OR = 0.95, 95% CI: 0.93–0.97, p = 0.001), Nail-biting behavior (OR = 0.93, 95% CI: 0.89–0.96, p = 0.001), and Hair-pulling behavior (OR = 0.95, 95% CI: 0.93–0.96, p = 0.001).

**Table 5:** Multivariable logistic regression analysis of factors associated with BFRBs and their subtypes.

Factor	BFRB OR (95% CI)	p-value	Skin-picking behavior OR (95% CI)	p-value	Nail-biting behavior OR (95% CI)	p-value	Hair-pulling behavior OR (95% CI)	p-value
Age	0.94 (0.93–0.96)	0.001*	0.95 (0.93–0.97)	0.001*	0.93 (0.89–0.96)	0.001*	0.95 (0.93–0.96)	0.001*
Female gender	1.17 (0.81–1.70)	0.392	1.15 (0.71–1.87)	0.562	0.53 (0.26–0.96)	0.048*	1.56 (1.01–2.42)	0.044*
Education	1.03 (0.78–1.37)	0.816	1.15 (0.79–1.68)	0.456	0.68 (0.38–1.21)	0.186	0.93 (0.67–1.28)	0.645

BFRBs, body-focused repetitive behaviors; OR, odds ratio; CI, confidence interval.

*P < 0.05 (significant).

Female gender was not a significant predictor of overall BFRBs (OR = 1.17, 95% CI: 0.81–1.70, p = 0.392) or Skin-picking behavior (OR = 1.15, 95% CI: 0.71–1.87, p = 0.562). However, females had significantly lower odds of Nail-biting behavior (OR = 0.53, 95% CI: 0.26–0.96, p = 0.048) and higher odds of Hair-pulling behavior (OR = 1.56, 95% CI: 1.01–2.42, p = 0.044). However, educational level was not associated with any of the outcomes, with all p-values > 0.05.

## Discussion

This study assessed the prevalence and characteristics of body-focused repetitive behaviors (BFRBs) among the general population of Saudi Arabia and found a relatively high overall prevalence of 29.1%. This prevalence is higher than several earlier international surveys that used comparable behavioral screening tools (11), suggesting that differences in methodology, study populations, or cultural factors may contribute to variability in reported rates. Although females showed higher crude prevalence than males (33.8% vs. 21.8%), this difference was partly influenced by the overrepresentation of women in the sample. Furthermore, multivariable analysis did not identify female gender as an independent predictor of overall repetitive behaviors or skin-picking; significant associations were observed only for hair-pulling (higher odds in females) and nail-biting (higher odds in males).

It is important to note that this study does not establish DSM-5 diagnoses. The behaviors captured reflect a spectrum of repetitive grooming habits, many of which may be benign or transient. The study-defined thresholds were used to identify individuals reporting higher frequency and impairment, but a formal clinical diagnosis requires structured psychiatric assessment. A subset of individuals meeting these criteria may nonetheless warrant further evaluation.

Consistent with earlier reports, Hair-pulling behavior and Skin-picking behavior were more prevalent among females, while Nail-biting behavior did not differ significantly between genders. Previous studies have similarly reported that females are disproportionately affected by Hair-pulling behavior and skin-picking disorders (3, 10). The higher frequency in women has been attributed to hormonal influences, emotion regulation difficulties, and greater reliance on maladaptive coping strategies in response to stress and anxiety (6).

The age distribution in our study revealed that BFRBs were most common in younger individuals, particularly those aged 18–20 years (47.2%), and gradually declined with age, reaching 10.1% in participants older than 50 years. This pattern aligns with prior evidence showing that BFRBs typically emerge in adolescence and often decrease in severity with advancing age (8, 9). The decline with age may reflect the acquisition of stronger impulse control, improved emotional regulation, or the impact of social conditioning that discourages visible repetitive behaviors (3).

Contrary to some reports linking higher educational attainment to prevalence of oral parafunctions and related behaviors (12), our study found no significant association between education and BFRB status. This suggests that these behaviors may be more strongly influenced by psychological and emotional factors than by formal education.

The association between BFRBs and psychiatric comorbidities has been well documented. Prior studies have established links with depression, anxiety, ADHD, and disorders of impulse control (7). Individuals with difficulties in emotional regulation or impulsivity are more likely to develop BFRBs as maladaptive coping mechanisms (3). Although we did not directly measure psychiatric diagnoses, our findings are consistent with these associations and highlight the importance of incorporating mental health screening in BFRB management.

Cultural context must also be considered. In Saudi Arabia, stigma surrounding mental and behavioral disorders may discourage individuals from seeking professional help. This is reflected in our data, where only 5.5% of affected participants reported consulting a healthcare provider. Similar findings have been noted in other regions, where lack of awareness and cultural attitudes contribute to underdiagnosis and underutilization of care (5).

The clinical implications of these findings are considerable. Early recognition and intervention are essential to prevent complications such as scarring, functional impairment, and psychosocial distress. Healthcare professionals, particularly in primary care, should maintain awareness of BFRBs, especially among young adults and females. Awareness campaigns and educational initiatives may help reduce stigma and encourage earlier help-seeking, as emphasized by prior recommendations (1, 2).

### Limitation

This study has limitations. Its cross-sectional design precludes causal inference, and reliance on self-reported online questionnaires may introduce bias compared with clinician-administered assessments. Furthermore, cultural influences on reporting and help-seeking were not examined in detail, which may shape both prevalence and treatment patterns. This study did not differentiate sex assigned at birth from gender identity and did not include race or ethnicity variables, which restricts demographic interpretation and generalizability.

### Conclusion and recommendation

In conclusion, BFRB-like behaviors defined by this study’s criteria were found to be common in the Saudi population, with nearly one-third of participants meeting thresholds for at least one repetitive grooming behavior. Younger individuals demonstrated the highest prevalence, while gender differences varied by behavior subtype rather than showing a uniform pattern. Despite reports of functional interference and physical consequences, only a small proportion of affected individuals sought medical attention. These findings highlight the urgent need for targeted awareness efforts, integration of behavioral-screening tools into primary care, and culturally sensitive interventions to improve recognition and support for individuals exhibiting high-frequency or impairing repetitive grooming behaviors.

## Data Availability

The raw data supporting the conclusions of this article will be made available by the authors, without undue reservation.
