# Correction: Prevalence of body-focused repetitive behaviors among the general population of Saudi Arabia: a cross-sectional study

**DOI:** 10.3389/fpsyt.2026.1842959

**Published:** 2026-04-08

**Authors:** Safiah A. Alamer, Hassan M. Alturaiki, Ali J. AlSaad, Ali M. Al Mousa, Amani A. Almutairi, Zahra S. Al-Sindi, Dalal M. Motabagani, Nouran D. AlShehri, Mariam M. Alshams

**Affiliations:** 1 Department of General Medicine, College of Medicine, King Faisal University, Al Ahsa, Saudi Arabia; 2 Department of Psychiatry, College of Medicine, King Faisal University, Al Ahsa, Saudi Arabia

**Keywords:** body-focused repetitive behaviors, hair-pulling behavior, nail-biting behavior, Saudi Arabia, skin-picking behavior

The funder “Deanship of Scientific Research, Vice Presidency for Graduate Studies and Scientific Research, King Faisal University, Saudi Arabia (Grant No. KFU251008) to Alamer SA” was erroneously omitted. The correct statement is:

“The author(s) declared that financial support was received for this work and/or its publication. This work was supported by the Deanship of Scientific Research, Vice Presidency for Graduate Studies and Scientific Research, King Faisal University, Saudi Arabia (Grant No. KFU251008) to Alamer SA”.

The original version of this article has been updated.

